# Supplementary material for: Chromothripsis during telomere crisis is independent of NHEJ, and consistent with a replicative origin
Source: Genome Res. 2019 May;29(5):737–49. doi: 10.1101/gr.240705.118 (PMC6499312; doi:10.1101/gr.240705.118)
Supplement: Supplemental Material [file supp_gr.240705.118_Supplemental_file_1.zip › contigs/annotated_contigs/DB114/contig.2.DB114_length_285_mean_cov_4.21052631579.docx]

**DB114_length_285_mean_cov_4.21052631579**

```
TGTCTGCTGCTAGCTTGTCTAGTTATTTTCATTATAATGTTAGGGTGTCGATTTTAGTTCTTTCCTGCTTTCTCCTGTGGGCATTTAGT
 >chr2:120379409-120379575 - E=9e-89
GCTATAAATTTCCCTCTAAACATTACTTCAGCTGTGTCCCAGAGATTCTGGTATGTTGTGTCTTTGTTCTCATTGGT|CATGA|CCATG
                                                                                   >chr
TTGGTCAGACTGGTCTCGAACTCCCGACCTGAGGTGATCCGCCTGCCTTGACCTCCCAATGTGCTGGGATTACAGGCATGAGCCACCAT
2:120378506-120378620 - E=2e-57
GCCCAGCCAACCCCTGCTTT
```
